# PSMA PET Validates Higher Rates of Metastatic Disease for European Association of Urology Biochemical Recurrence Risk Groups: An International Multicenter Study

**DOI:** 10.2967/jnumed.121.262821

**Published:** 2022-01

**Authors:** Justin Ferdinandus, Wolfgang P. Fendler, Andrea Farolfi, Samuel Washington, Osama Mohamad, Miguel H. Pampaloni, Peter J.H. Scott, Melissa Rodnick, Benjamin L. Viglianti, Matthias Eiber, Ken Herrmann, Johannes Czernin, Wesley R. Armstrong, Jeremie Calais, Thomas A. Hope, Morand Piert

**Affiliations:** 1 Department of Nuclear Medicine, University of Duisburg–Essen and German Cancer Consortium–University Hospital Essen, Essen, Germany;; 2 Ahmanson Translational Imaging Division, Department of Molecular and Medical Pharmacology, UCLA, Los Angeles, California;; 3 Division of Nuclear Medicine, IRCCS Azienda Ospedaliero–Universitaria di Bologna, Bologna, Italy;; 4 Department of Urology, University of California San Francisco, San Francisco, California;; 5 Department of Epidemiology and Biostatistics, University of California San Francisco, San Francisco, California;; 6 Department of Radiation Oncology, University of California San Francisco, San Francisco, California;; 7 Department of Radiology and Biomedical Imaging, University of California San Francisco, San Francisco, California;; 8 Department of Radiology, University of Michigan, Ann Arbor, Michigan; and; 9 Department of Nuclear Medicine, Klinikum rechts der Isar, Technical University Munich, Munich, Germany

**Keywords:** EAU, risk score, prostate cancer, prostate specific membrane antigen, PSMA

## Abstract

The European Association of Urology (EAU) prostate cancer guidelines panel recommends risk groups for biochemical recurrence (BCR) of prostate cancer to identify men at high risk of progression or metastatic disease. The rapidly growing availability of PSMA-directed PET imaging will impact prostate cancer staging. We determined the rates of local and metastatic disease in BCR and biochemical persistence (BCP) of prostate cancer stratified by EAU BCR risk groups and BCP. **Methods:** Patients with BCR or BCP were enrolled under the same prospective clinical trial protocol conducted at 3 sites (*n* = 1,777 [91%]: UCLA, *n* = 662 [NCT02940262]; University of California San Francisco, *n* = 508 [NCT03353740]; University of Michigan, *n* = 607 [NCT03396874]); 183 patients with BCP from the Universities of Essen, Bologna, and Munich were included retrospectively. Patients with BCR had to have sufficient data to determine the EAU risk score. Multivariate, binomial logistic regression models were applied to assess independent predictors of M1 disease. **Results:** In total, 1,960 patients were included. Post–radical prostatectomy EAU BCR low-risk, EAU BCR high-risk, and BCP groups yielded distant metastatic (M1) detection in 43 of 176 (24%), 342 of 931 (37%), and 154 of 386 (40%) patients. For postradiotherapy EAU BCR low-risk and EAU BCR high-risk groups, the M1 detection rate was 113 of 309 (37%) and 110 of 158 (70%), respectively. BCP, high-risk BCR, and higher levels of serum prostate-specific antigen were significantly associated with PSMA PET M1 disease in multivariate regression analysis. PSMA PET revealed no disease in 25% and locoregional-only disease in 33% of patients with post–radical prostatectomy or postradiotherapy EAU BCR high risk. **Conclusion:** Our findings support the new EAU classification; EAU BCR high-risk groups have higher rates of metastatic disease on PSMA PET than do the low-risk groups. Discordant subgroups, including metastatic disease in low-risk patients and no disease in high-risk patients, warrant inclusion of PSMA PET stage to refine risk assessment.

## Visual Abstract

**Figure:**
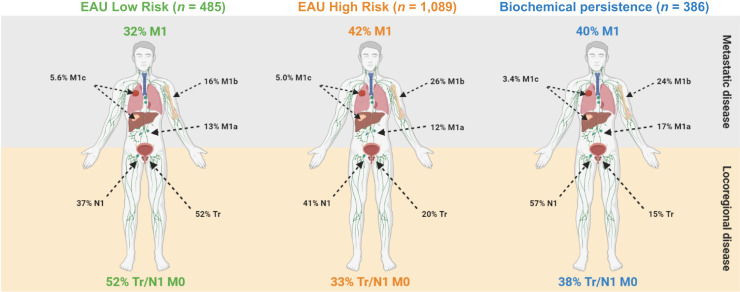

After primary curative-intent treatment for prostate cancer with radical prostatectomy (RP) or radiotherapy, approximately 1 of 4 men experience biochemical recurrence (BCR) (*1*).

The incidence and outcomes of BCR are variable. A novel European Association of Urology (EAU) risk-scoring system combines prostate-specific antigen (PSA) doubling time, Gleason score, and interval from primary therapy to biochemical failure to identify patients at high risk for metastases and early disease progression (*2*). PSA biochemical persistence (BCP) was described as a different pattern of relapse, which is associated with worse oncologic outcomes and was therefore not stratified into risk groups (*3*).

Tilki et al. validated the EAU BCR risk score using survival data from an extensive dataset of post-RP patients from their center. Metastatic progression-free and overall survival were significantly different; however, the prognostic accuracy for metastasis-free survival (concordance index, 0.67) or disease-specific survival (concordance index, 0.69) was moderate, warranting further refinement of this classification (*4*).

PSMA-targeted PET has demonstrated high detection rates and accuracy for the localization of prostate cancer metastases (*5*). The improved accuracy of PSMA PET, along with the impact on management, led to its inclusion in the EAU guidelines and to Food and Drug Administration approval for imaging primary disease and BCR (*6*,*7*). Several trials evaluating the potential of PSMA PET–guided therapy to achieve an improved outcome are currently under way or were recently published (*8*,*9*). PSMA PET disease extent was associated with time to progression in patient candidates for salvage radiotherapy and may thus offer independent prognostic value at BCR and BCP (*10*).

The aim of this study was to assess disease extent in patients with EAU BCR high risk, EAU BCR low risk, and BCP using PSMA PET to identify subgroups of undetectable (T0N0M0), locoregional (Tr/N1), or distant metastatic (M1) disease.

## MATERIALS AND METHODS

This was a multicentric, single-arm analysis of patients with BCR or BCP of PSA after curative treatment of prostate cancer. BCR was defined as a PSA of at least 0.2 ng/mL measured more than 6 wk after RP or a PSA rise by at least 2 ng/mL above nadir after radiation therapy. BCP was defined as a PSA nadir of more than 0.1 ng/mL within 12 wk after RP. The final database consisted of 1,960 patients with either BCR (*n* = 1,574) or BCP (*n* = 386). Most patients were enrolled under the same prospective clinical trial protocol conducted at 3 sites (*n* = 1,777[91%]: UCLA, *n* = 662 [NCT02940262]; University of California San Francisco, *n* = 508 [NCT03353740]; University of Michigan, *n* = 607 [NCT03396874]); 183 patients with BCP from the Universities of Essen, Bologna, and Munich were included retrospectively. In total, 587 of 1,960 (30%) patients have been reported previously (*5*,*6*,*11*). The study was approved by institutional review boards at each site.

**FIGURE 1.**
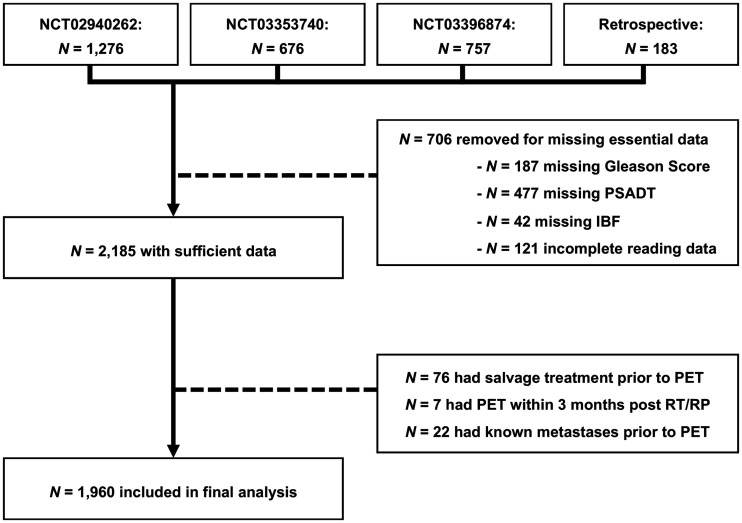
Study flowchart. IBF = interval from primary therapy to biochemical failure; PSADT = PSA doubling time; RT = radiotherapy.

Patients were eligible if they had a history of histopathology-proven prostate adenocarcinoma and BCR or BCP after curative-intent radiotherapy or RP. Further, BCR patients had to have sufficient data to determine risk group: PSA doubling time and Gleason score for recurrence after RP, interval from primary therapy to biochemical failure, and Gleason score after radiotherapy. Patients had to have complete reading data. Patients with known metastases before PSMA PET, prior salvage treatment, or PSMA PET within 3 mo after curative treatment were not eligible for this analysis. A flowchart for patient inclusion is shown in Figure 1.

Detailed imaging procedures were reported previously (*5*), and scans were acquired in accordance with the international guideline (*12*). In brief, whole-body PET was acquired from skull to mid thighs. PET was performed as hybrid imaging with CT or MRI based on availability and contraindications. For PET/CT, a diagnostic contrast-enhanced CT scan was obtained before the PET scan. For PET/MRI, an abbreviated pelvic PET/MRI scan was obtained following a whole-body protocol after the PET scan. PSMA PET findings were interpreted using PROMISE (Prostate Cancer Molecular Imaging Standardized Evaluation) criteria (*13*).

Descriptive statistics were used to report patient characteristics and disease extent. Multivariate, binomial logistic regression models were applied to assess independent predictors of M1 disease. Analyses were performed using R, version 3.4.0 (R Foundation for Statistical Computing). Figure parts were created using BioRender Software.

## RESULTS

**FIGURE 2.**
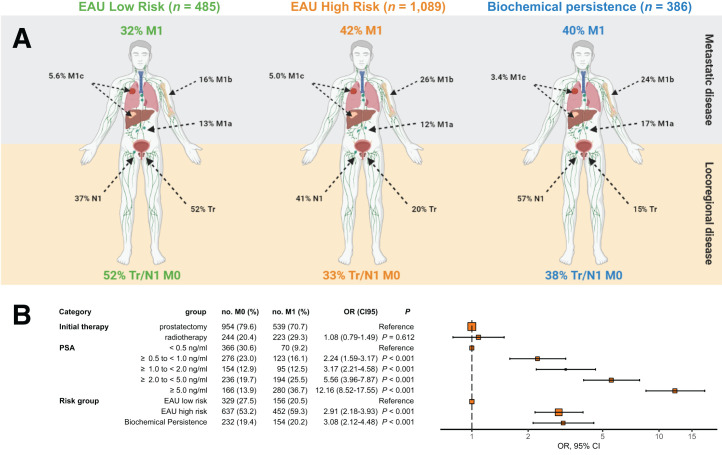
PET disease extent in EAU BCR low-risk patients, EAU BCR high-risk patients, and BCP patients (A) and predictors of PET M1 disease (B). OR = odds ratio.

Table 1 lists patient characteristics and PSMA PET stage. The median PSA serum level at the time of PSMA PET was 1.76 ng/mL (interquartile range [IQR], 4.28 ng/mL). PSA values differed after RP (median, 1.0 ng/mL; IQR, 2.4 ng/mL) versus after radiotherapy (median, 5.1 ng/mL; IQR, 6.4 ng/mL). In total, 1,493 (76%) patients received primary RP and 467 (24%) patients received primary radiotherapy. More than 60% of patients in the post-RP group had a PSA level of less than 2.0 ng/mL, whereas—also because of a difference in BCR definition—most of the postradiotherapy patients had a PSA level of at least 2 ng/mL. The median time since initial therapy was the longest in the respective EAU BCR low-risk groups (post-RP, 9.6 mo [IQR, 7.4 mo]; postradiotherapy, 7.4 mo [IQR, 6.9 mo]). PSMA PET localized disease in 1,515 of 1,960 (77%) patients. Figure 2A shows the PSMA PET–detected disease extent separate for EAU BCR risk groups and BCP.

**TABLE 1:** Patient Characteristics and PSMA PET Stages

	RP			Radiotherapy	
Characteristic	EAU BCR low risk (*n* = 176)	EAU BCR high risk (*n* = 931)	BCP (*n* = 386)	EAU BCR low risk (*n* = 309)	EAU BCR high risk (*n* = 158)
Age (y)	71 [9.3]	69 [9.1]	70 [12]	73 [9.6]	72 [9.1]
PSA ( ng/mL)					
<0.5	60 (34.1%)	302 (32.4%)	69 (17.9%)	3 (1.0%)	2 (1.3%)
≥0.5 to <1.0	38 (21.6%)	178 (19.1%)	175 (45.3%)	4 (1.3%)	4 (2.5%)
≥1.0 to <2.0	20 (11.4%)	174 (18.7%)	43 (11.1%)	7 (2.3%)	5 (3.2%)
≥2.0 to <5.0	34 (19.3%)	159 (17.1%)	41 (10.6%)	134 (43.4%)	62 (39.2%)
≥5.0	24 (13.6%)	118 (12.7%)	58 (15.0%)	161 (52.1%)	85 (53.8%)
PSA doubling time (mo)	20 [18]	4.2 [5.2]	4.5 [5.8]	8.5 [11]	4.1 [5.7]
Gleason score					
6	30 (17.0%)	42 (4.5%)	17 (4.4%)	97 (31.4%)	2 (1.3%)
7	146 (83.0%)	507 (54.5%)	168 (43.5%)	212 (68.6%)	27 (17.1%)
8	—	168 (18.0%)	79 (20.5%)	—	55 (34.8%)
9–10	—	214 (23.0%)	122 (31.6%)	—	74 (46.8%)
IBF (mo)	83 [78]	44 [51]	34 [55]	88 [84]	41 [65]
Adjuvant RT after RP					
Adjuvant RT	50 (28.4%)	368 (39.5%)	78 (20.2%)	—	—
No adjuvant RT	126 (71.6%)	563 (60.5%)	308 (79.8%)	309 (100%)	158 (100%)
PSMA PET stage					
T0N0M0 (no disease)	58 (33.0%)	275 (29.5%)	85 (22.0%)	20 (6.5%)	7 (4.4%)
Tr/N1M0 (locoregional)	75 (42.6%)	314 (33.7%)	147 (38.1%)	176 (57.0%)	41 (25.9%)
Any M1 (metastatic)	43 (24.4%)	342 (36.7%)	154 (39.9%)	113 (36.6%)	110 (69.6%)
M1 group					
M1a only	13 (7.4%)	102 (11.0%)	53 (13.7%)	49 (15.9%)	30 (19.0%)
Any M1b*	19 (10.8%)	201 (21.6%)	88 (22.8%)	48 (15.5%)	65 (41.1%)
Any M1c	11 (6.2%)	39 (4.2%)	13 (3.4%)	16 (5.2%)	15 (9.5%)
No. of M1 regions					
0	133 (75.6%)	589 (63.3%)	232 (60.1%)	196 (63.4%)	48 (30.4%)
1–2	5 (2.8%)	55 (5.9%)	44 (11.4%)	11 (3.6%)	18 (11.4%)
≥3	38 (21.6%)	287 (30.8%)	110 (28.5%)	102 (33.0%)	92 (58.2%)

*Not including M1c.

IBF = interval from primary therapy to biochemical failure; RT = radiotherapy.

Qualitative data are number followed by percentage in parentheses; continuous data are median followed by IQR in brackets. PSMA stages are according to PROMISE criteria (*13*).

PSMA PET revealed M1 disease within the post-RP group in 43 of 176 (24%), 342 of 931 (37%), and 154 of 386 (40%) EAU BCR low-risk, EAU BCR high-risk, and BCP patients, respectively. Within the postradiotherapy group, M1 disease was detected in 113 of 309 (37%) and 110 of 158 (70%) EAU BCR low- and high-risk patients, respectively. Bone metastases were detected in 19 of 176 (11%), 201 of 931 (37%), and 88 of 386 (23%) post-RP EAU BCR low-risk, EAU BCR high-risk, and BCP subgroups, respectively, and in 16 of 309 (5%) and 15 of 158 (10%) postradiotherapy EAU BCR low- and high-risk subgroups, respectively. Visceral metastases were detected in 3%–6% of patients in the post-RP subgroups and in 16 of 309 (5%) and 15 of 158 (10%) in the postradiotherapy EAU BCR low- and high-risk subgroups, respectively. The number of involved regions differed among the different risk groups. Three or more involved metastatic regions were detected in 38 of 176 (22%), 287 of 931 (31%), and 110 of 386 (29%) post-RP EAU BCR low-risk, EAU BCR high-risk, and BCP patients, respectively, as well as 102 of 309 (33%) and 92 of 158 (58%) postradiotherapy EAU BCR low- and high-risk patients, respectively.

PSMA PET revealed no disease in 58 of 176 (33%), 275 of 931 (30%), and 85 of 386 (22%) post-RP EAU BCR low-risk, EAU BCR high-risk, and BCP subgroups. Postradiotherapy subgroups were PET-negative in 20 of 309 (7%) EAU BCR low-risk patients and 7 of 158 (4%) EAU BCR high-risk patients, respectively.

Figure 2B shows a Forest plot for odds ratios derived from multivariate regression. Higher PSA levels, EAU BCR high risk (odds ratio, 2.91; 95% CI, 2.18–3.93), and BCP (odds ratio, 3.08; 95% CI, 2.12–4.48) were significantly associated with PSMA PET M1 disease, whereas type of initial therapy was not.

## DISCUSSION

^68^Ga-PSMA-11 and ^18^F-DCFPyL PET were recently approved by the Food and Drug Administration on the basis of high accuracy for prostate cancer staging (*5*,*11*,*14*). Approval of PSMA-ligand PET is soon expected to enable broad availability for staging of BCR or BCP. Our findings present a detailed map of disease extent in the EAU BCR risk groups or BCP. The observed intra- and intergroup heterogeneities in PET stage come with important implications for the EAU classification system.

First, PSMA PET stratified EAU BCR or BCP groups into relevant subgroups with undetectable, locoregional, or distant metastatic disease. After RP, about one third of patients was stratified into each of these 3 subgroups, with somewhat higher rates for metastatic disease in the BCR high-risk or BCP patients. We present a single-time-point assessment; however, PET stage was associated with time to progression in a previous prospective study on BCR (*10*).

Recently, Dong et al. noted in a pooled analysis of 145 patients after RP or radiotherapy that the EAU BCR high-risk group was associated with a higher PSMA PET positivity rate (*15*). In this study, we assessed patients after RP or radiotherapy separately and found similar rates for PET positivity but higher rates for metastatic disease in patients with EAU BCR high risk as compared with low risk. In addition, PSMA PET identified subgroups with discordant findings for EAU BCR risk label versus PET stage: 30% of post-RP EAU BCR high-risk patients had undetectable disease, whereas 24% of low-risk patients had metastatic disease, including 11% with bone metastases and 6% with visceral metastases. Discordant findings together with previous evidence by Emmett et al. (*10*) indicate that PSMA PET has additional prognostic value and should be considered for future risk assessment.

Second, disease extent detected by PSMA PET was higher in postradiotherapy patients than in RP patients: Postradiotherapy EAU BCR low-risk patients yield PSMA PET M1 rates similar to post-RP high-risk or BCP patients. Strikingly, more than two thirds of postradiotherapy high-risk patients had metastases, including bone metastases in 31.4% and visceral metastases in 14.5%. In patients with EAU BCR high risk, the incidence of M1 after radiotherapy was nearly twice that after RP; the rate of M1 visceral disease was more than 2 times higher. Because of different BCR definitions, this increase can be attributed to higher PSA values after radiotherapy (median, 5.1 ng/mL; IQR, 6.4 ng/mL) than after RP (median, 1.0 ng/mL; IQR, 2.4 ng/mL). Accordingly, initial therapy was not a significant predictor of metastatic disease in multivariate regression analysis with PSA levels included. We assume that the heterogeneous PSMA PET disease extent reflected clinical reality, that is, the postradiotherapy or post-RP EAU BCR risk groups will likely present different outcomes despite sharing the same risk label. To account for these differences, PSA level as well as RP- or radiotherapy-specific risk group nomenclature should be considered for risk assessment. We confirm a previously reported association of PSA with PSMA PET M1 disease. PSA level was a stronger predictor of the presence of M1 disease than was EAU BCR risk group. BCR or BCP states are defined using PSA kinetics without specific inclusion of individual PSA values. However, in the transition phase with limited availability of PSMA PET, PSA level will help identify patients at high risk who may benefit from PSMA PET staging.

## CONCLUSION

We demonstrate that men with high-risk BCR according to the EAU prostate cancer guidelines panel and BCP have higher rates of metastatic disease. Discordant subgroups, including metastatic disease in low-risk patients and no disease in high-risk patients, warrant inclusion of PSMA PET stage to refine risk assessment.

## DISCLOSURE

Justin Ferdinandus has received fees from Eisai outside the submitted work. Wolfgang Fendler reports fees from Calyx (consultant), RadioMedix (image reviewer), Bayer (member of speakers’ bureau), and Parexel (image reviewer) outside the submitted work. Matthias Eiber reports personal fees from Blue Earth Diagnostics, Progenics Pharmaceuticals, Amgen, Parexel, Bayer, and Point Biopharma; research support from Blue Earth Diagnostics; and a patent application for rhPSMA outside the submitted work. Ken Herrmann reports personal fees from Bayer, Sofie Biosciences, SIRTEX, Adacap, Curium, Endocyte, BTG, IPSEN, Siemens Healthineers, GE Healthcare, Amgen, Novartis, and ymabs; other fees from Sofie Biosciences; nonfinancial support from ABX; and grants from BTG, outside the submitted work. Johannes Czernin is a cofounder and holds equity in Sofie Biosciences and Trethera Therapeutics and is a consultant for Blue Earth Diagnostics, Progenics Radiopharmaceuticals, and Radiomedix, outside the submitted work. Thomas Hope is on a consultancy or advisory board for Curium and Ipsen, performs research for Clovis Oncology and Philips, and is a trial participant for Novartis and AAA. Intellectual property patented by the University of California has been licensed to Sofie Biosciences and Trethera Therapeutics. This intellectual property was not used in the current study. No other potential conflict of interest relevant to this article was reported.

## Notes

KEY POINTS **QUESTION:** Does the new EAU risk classification identify distinct patterns of disease spread in PSMA PET? **PERTINENT FINDINGS:** In this multicenter, international study including 1,960 men with BCR or BCP of prostate cancer, we found that high-risk EAU BCR and BCP was significantly associated with a higher risk of metastatic disease on PSMA PET. However, PSMA PET also found patients with discordant patterns, that is, no detected disease in high-risk patients and metastatic disease in low-risk patients. **IMPLICATIONS FOR PATIENT CARE:** PSMA PET validates the novel EAU BCR risk classification. In addition, it may further refine risk assessment in this cohort.
